# A Rare Presentation of Hepatitis A Infection with Extrahepatic Manifestations

**DOI:** 10.1155/2014/286914

**Published:** 2014-09-14

**Authors:** Geetika Bhatt, Varrinder S. Sandhu, Charlene K. Mitchell

**Affiliations:** ^1^Department of Internal Medicine, University of Louisville, 529 South Jackson Street, Louisville, KY 40202, USA; ^2^Medicine and Pediatrics, Department of Internal Medicine, University of Louisville, 529 South Jackson Street, Louisville, KY 40202, USA

## Abstract

Hepatitis A has a variety of associated extrahepatic manifestations that clinicians should be aware of for early diagnosis and treatment. We report a unique case of hepatitis A presenting with multiple extrahepatic manifestations not previously described in a single patient. A 34-year-old male presented with sudden onset of left sided facial pain, swelling, and skin rash, with diffuse body pains and muscle weakness, and was found to be positive for hepatitis A immunoglobulin M (IgM). He was initially started on antibiotics for concerns of bacterial parotitis but did not show any improvement. A punch biopsy of his mandibular rash and swelling was done which showed lymphohistiocytic infiltration with a few eosinophils. A trial of prednisone resulted in improvement of his symptoms. Clinicians should be aware to look for hepatitis A infection in a patient with atypical clinical picture causing a widespread systemic inflammatory response. Treatment with prednisone may result in resolution.

## 1. Introduction

Hepatitis A virus infection is uncommon in the United States. A variety of extrahepatic manifestations that clinicians should be aware of have been described that appear to be immune mediated. We report a unique case of hepatitis A presenting with multiple extrahepatic manifestations not previously described in a single patient.

## 2. Case

A 34-year-old male presented with sudden onset of left sided facial pain, swelling, and skin rash, with diffuse body pains, muscle weakness, numbness in his left arm, and an otherwise asymptomatic clear penile discharge. There were no fevers, chills, nausea, vomiting, or diarrhea. Exposure history was negative for travel, ticks, dog bites, or new sexual partners. Past medical history and review of systems were unremarkable, and he was not taking any medications. Physical examination was notable for normal conjunctiva, a moist oropharynx with poor dentition; a soft, nontender abdomen without hepatosplenomegaly; a nonfluctuant, indurated 2 × 2 centimeters (cm) swelling over the angle of his left mandible without well-defined margins, with an overlying 2 × 3 cm, pink, salmon colored, maculopapular rash; similar skin lesions present on the chest wall, left elbow, and right wrist; decreased sensation of light touch in the left hand and forearm; and a clear viscous penile discharge. The remainder of the examination, including joints, was unremarkable.

Initial laboratory data showed leukocytosis, hyperkalemia, lactic acidosis, and elevated creatinine phosphokinase and aspartate aminotransferase which was secondary to rhabdomyolysis (Table 1). Urinalysis was positive for blood without red blood cells, and with hyaline casts present indicative of myoglobinuria consistent with his rhabdomyolysis. The cause was thought to be secondary to sepsis; he was admitted to the intensive care unit (ICU) and treated with aggressive intravenous fluid therapy and empiric antibiotic coverage.

The differential diagnoses included infectious etiologies of bacterial endocarditis, disseminated gonococci, meningococci, tick borne diseases like Ehrlichiosis, West Nile fever, and Rocky Mountain spotted fever, and vasculitis. A vasculitic and infectious disease workup was initiated (Table 2). Computed tomography (CT) scan of his chest, abdomen, and pelvis was normal except for a slightly enlarged liver with slightly decreased density but without cirrhotic appearance. After adequate fluid resuscitation, his rhabdomyolysis and lactic acidosis improved, and he was transferred to general medicine on day 2.

CT scan was done for enlarging mandibular swelling, which showed asymmetric, left parotid gland inflammation without signs of abscess formation. Infectious disease consult was obtained and his antibiotics were changed to vancomycin and clindamycin for bacterial parotitis. By day 4, patient's aspartate aminotransferase returned to normal but his alanine aminotransferase remained mildly elevated. On day 6, he developed diffuse joint pains and increased right upper quadrant abdominal pain with rise in alanine aminotransferase. Ultrasound of his liver showed hepatomegaly with periportal edema consistent with acute hepatitis. His infectious and rheumatological tests all were normal, except his hepatitis panel, which was positive for hepatitis A virus IgM (HAV IgM) only.

**Figure 1:**
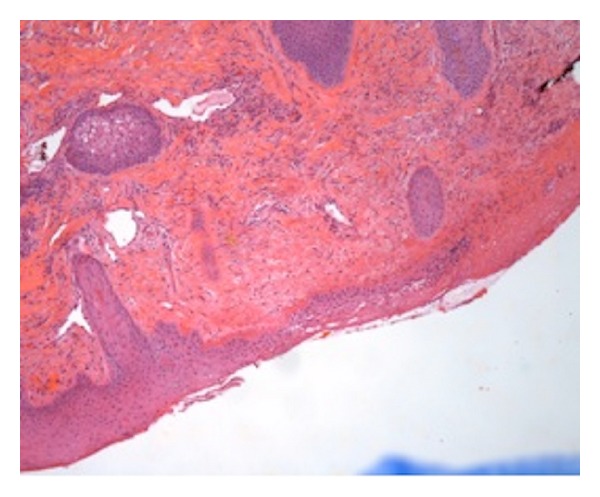
Punch biopsy of the patient's swelling showing the area of ulceration with dermal superficial and deep perivascular and periadnexal chronic inflammation with eosinophils.

His joint pains, rash, and mandibular swelling persisted despite adequate antibiotic treatment. A punch biopsy of his mandibular rash and swelling was done, which showed lymphohistiocytic infiltration with a few eosinophils and rare neutrophils (Figure 1). Given his negative infectious workup except for hepatitis A IgM antibody, the antibiotics were discontinued. False positive result for hepatitis IgM was possible but unlikely without other viral illness and a negative rheumatoid factor. The patient was placed on prednisone empirically as review of the literature indicated that all of his symptoms could be hepatic and extrahepatic manifestations of hepatitis A infection via an autoimmune mechanism due to immune-complex deposition. Upon treatment with prednisone, his diffuse joint and muscle pain improved; his parotitis decreased in size by day 3 of prednisone, and most of his symptoms had disappeared by day 4. Repeat testing of hepatitis A antibodies showed decreasing IgM titers. He was discharged home with a long taper of prednisone.

## 3. Discussion

Hepatitis A disease in the United States has declined in incidence to 1 case in 100,000 [1]. Patients develop abrupt onset of prodromal symptoms of malaise, joint pain (11%), right upper quadrant pain, and evanescent rash (14%) even weeks before developing jaundice (40% to 70%) in acute infection [2–4]. Atypical manifestations of prolonged cholestasis, relapsing hepatitis, and extrahepatic involvement, which are rare unlike in hepatitis B or hepatitis C, may be present. Extrahepatic manifestations may include acute kidney injury, urticarial and maculopapular rash, polymyositis, arthralgias, and suppurative parotitis that can be seen in both prodrome and acute infection [5, 6]. Polymyositis can result in rhabdomyolysis, as seen in our patient with elevated creatinine phosphokinase and myoglobinuria [7].

Many cases of acute kidney injury associated with hepatitis A infection have been described in endemic areas [8]. Causes of acute kidney injury in hepatitis A infection include myoglobinuria secondary to polymyositis from infiltration by inflammatory cells and acute necrosis of the muscle fibers; circulating immune-complex mediated nephritis; and cryoglobulinemia or by direct viral invasion [9–13]. Kidney biopsies in cases of renal failure in patients with hepatitis A have shown interstitial nephritis, acute tubular necrosis, and some with no renal pathology. Spontaneous resolution of acute kidney injury in interstitial nephritis has also been reported [14, 15]. Renal failure secondary to fulminant hepatitis improved in large doses of methylprednisolone, thus indicating an immune mediated reaction [16].

Acute hepatitis can be associated with evanescent rash in up to 14% of the cases, but it is usually a transient phenomenon. Spontaneous resolution occurs once the infection has cleared [4]. Erythematous maculopapular rash is secondary to cutaneous vasculitis from deposition of immune complexes in the skin but may be due to cryoglobulinemia [17–19].

Arthritis has a predilection for lower extremities and spontaneous resolution may occur [4]. Hepatitis A has been known to even mimic Adult Onset Still's Disease, characterized by fever, arthralgia, rash, and leukocytosis (Table 3) [20]. Two cases have been reported in the literature of Adult Onset Still's Disease with hepatitis A virus being the causative agent [21, 22]. It has been postulated that T helper 1 cytokines mediate the association between acute hepatitis A infection and Adult Onset Still's Disease [20]. Treatment with large doses of prednisone resulted in improvement of symptoms as in our patient.

A variety of hematological abnormalities can be observed in acute hepatitis A. Hemolysis occurred in 10 patients out of 256 patients with acute hepatitis A hospitalized in Tennessee in 1994-1995 [23]. It can be precipitated by viral infection in patients with glucose-6-phosphate dehydrogenase deficiency or can be autoimmune. Other hematological abnormalities like autoimmune thrombocytopenic purpura, aplastic anemia, pure red cell aplasia, and decreased red cell survival in the absence of red cell abnormality can be observed [24]. Our patient did not have a history of glucose-6-phosphate dehydrogenase deficiency and did not have evidence of hemolysis during his hospitalization.

Diagnosis of hepatitis A is made by serological testing of IgM antibody in the clinical setting of acute infection. A positive test is considered false positive in a patient without clinical criteria, which can be varied from mild prodromal symptoms to fulminant hepatitis with or without extrahepatic manifestations [25]. False positive IgM tests have been reported with concomitant viral illnesses or a positive rheumatoid factor [26]. It may indicate hepatitis A virus exposure or dormant viral infection [25]. However in our patient, the right upper quadrant pain, hepatomegaly with periportal edema, evanescent rash, arthralgias, rhabdomyolysis, and parotitis were clinical manifestations of hepatitis A infection that resolved with normalization of hepatitis A IgM titers and treatment with prednisone.

## 4. Conclusion

Unlike hepatitis B and hepatitis C, extrahepatic manifestations are rare in hepatitis A. Clinicians should be aware to look for hepatitis A infection in a patient with atypical clinical picture. Extrahepatic manifestations are due to deposition of immune complexes in various organs causing a widespread systemic inflammatory response. Treatment with prednisone may result in resolution of these extrahepatic manifestations.

## Figures and Tables

**Table 1:** Laboratory data on admission and hospital day 6.

Variable	Reference range (adults)	On admission	Hospital day 6
Hemoglobin (g/dL)	13.7–17.5 (males)	15.5	14.2
Hematocrit (%)	40.1–51.0 (males)	46.7	43.1
White-cell count (per mm^3^)	4.1–10.8	**25,900**	**11,900**
White-cell differential count			
Neutrophils (%)	34.0–69.5	**82.7**	**70.8**
Immature granulocytes (%)	0–0.7	**0.9**	**1.3**
Lymphocytes (%)	20.0–53.0	**5.8**	14.3
Monocytes (%)	5.0–12.5	10.5	8.5
Eosinophils (%)	0.7–6.0	**0.0**	4.7
Basophils (%)	0.0–2.0	0.1	0.4
Platelet count (per mm^3^)	140,000–370,000	266,000	223,000
Sodium (mmol/L)	137–145	**133**	139
Potassium (mmol/L)	3.5–5.1	**7.2**	4.5
Chloride (mmol/L)	100–108	**99**	**95**
Bicarbonate (mmol/L)	22–30	**19**	28
Blood urea nitrogen (mg/dL)	7–20	**24**	14
Creatinine (mg/dL)	0.7–1.4	1.3	0.8
Alkaline phosphatase (U/L)	38–162	53	62
Aspartate aminotransferase (U/L)	10–50	**159**	**51**
Alanine aminotransferase (U/L)	20–70	**75**	**130**
Total bilirubin (mg/dL)	0.2–1.0	0.3	0.4
Lactate dehydrogenase (U/L)	310–620	**1490**	
Creatinine phosphokinase (U/L)	55–170	**10686**	
C-reactive protein (mg/dL)	0.00–0.49	**3.47**	**0.73**
Cortisol (mcg/dL)	4.5–22.7 (morning)	**42.20**	
Erythrocyte sedimentation rate	0–15 mm/hour	1	13
Lactic acid (mmol/L)	0.9–1.7	**6.0**	0.9

**Table 2:** Serological testing.

Serology	Result
Virology	
HIV	Negative
Herpes simplex viruses 1 and 2 (PCR)	Negative
Epstein Barr virus	Negative
Varicella zoster (IgG)	Immune
Mumps (IgM, IgG)	Negative

Tick borne panel	
Ehrlichiosis (IgM, IgG)	Negative
Rocky Mountain spotted fever (IgM, IgG)	Negative
Lyme's disease (IgM, IgG)	Negative
Anaplasma (IgM, IgG)	Negative

Hepatitis panel	
Hepatitis A (IgM, IgG)	**IgM-reactive**
	IgG-negative
Hepatitis B (HBsAg)	Negative
Hepatitis C	Negative

Vasculitis	
Rheumatoid factor	8.8
	(normal 0.0–11.9 IU/mL)
Antinuclear antibody	Negative

Rapid plasma reagin	Nonreactive

Toxoplasma	0.0

*Chlamydia trachomatis* DNA	Not detected

*Neisseria gonorrhoeae* DNA	Not detected

**Table 3:** Yamaguchi criteria for diagnosis of Adult Onset Still's Disease∗.

Major criteria	Minor criteria
Fever (>39°C) lasting for >1 week	Sore throat
Arthralgias/arthritis >2 weeks	Lymphadenopathy
Salmon colored rash on trunk or extremities during febrile episodes	Hepatomegaly or splenomegaly
Leukocytosis (>10,000/dL) with 80% granulocytes)	Abnormal liver function tests
	Negative antinuclear antibody and rheumatoid factor

*The presence of any other infections precludes the diagnosis of Adult Onset Still's Disease.

## References

[1] Daniels D, Grytdal S, Wasley A (2009). Surveillance for acute viral hepatitis—united States, 2007. *MMWR Surveillance Summaries*.

[2] Alarcon GS, Townes AS (1973). Arthritis in viral hepatitis. Report of two cases and review of the literature. *Johns Hopkins Medical Journal*.

[3] Lednar WM, Lemon SM, Kirkpatrick JW, Redfield RR, Fields ML, Kelley PW (1985). Frequency of illness associated with epidemic hepatitis A virus infections in adults. *American Journal of Epidemiology*.

[4] Schiff ER (1992). Atypical clinical manifestations of hepatitis A. *Vaccine*.

[5] Routenberg JA, Dienstag JL, Harrison WO (1979). Foodborne outbreak of hepatitis A: clinical and laboratory features of acute and protracted illness. *The American Journal of the Medical Sciences*.

[6] Franczak T, Matysiak W (1988). Suppurative parotitis in a child with hepatitis A. *Wiadomosci Lekarskie*.

[7] Ann SH, An GH, Lee SY (2009). A case of rhabdomyolysis during hospitalization for acute hepatitis A. *The Korean Journal of Hepatology*.

[8] Kim SE, Kim SJ, Kim HS (2006). Two cases of acute renal failure associated with non-fulminant acute hepatitis A. *The Korean Journal of Gastroenterology*.

[9] Vaboe A-L, Leh S, Forslund T (2002). Interstitial nephritis, acute renal failure in a patient with non-fulminant hepatitis A infection. *Clinical Nephrology*.

[10] Zikos D, Grewal KS, Craig K, Cheng J-C, Peterson DR, Fisher KA (1995). Nephrotic syndrome and acute renal failure associated with hepatitis A virus infection. *American Journal of Gastroenterology*.

[11] Cheema SR, Arif F, Charney D, Meisels IS, Cheema SS (2004). IgA-dominant glomerulonephritis associated with hepatitis A. *Clinical Nephrology*.

[12] Chio F, Bakir AA (1992). Acute renal failure in hepatitis A. *International Journal of Artificial Organs*.

[13] Aggarwal SP, Khurana SB, Sabharwal BD (1996). Hepatitis A associated with myoglobinuria. *Indian Journal of Gastroenterology*.

[14] Jung YJ, Kim W, Jeong JB (2010). Clinical features of acute renal failure associated with hepatitis A virus infection. *Journal of Viral Hepatitis*.

[15] Geltner D, Naot Y, Zimhoni O, Gorbach S, Bar-Khayim Y (1992). Acute oliguric renal failure complicating type A nonfulminant viral hepatitis: a case presentation and review of the literature. *Journal of Clinical Gastroenterology*.

[16] Abe M, Kaizu K, Matsumoto K (2007). A case report of acute renal failure and fulminant hepatitis associated with edaravone administration in a cerebral infarction patient. *Therapeutic Apheresis and Dialysis*.

[17] Inman RD, Hodge M, Johnston MEA, Wright J, Heathcote J (1986). Arthritis, vasculitis, and cryoglobulinemia associated with relapsing hepatitis A virus infection. *Annals of Internal Medicine*.

[18] Dan M, Yaniv R (1990). Cholestatic hepatitis, cutaneous vasculitis, and vascular deposits of immunoglobulin M and complement associated with hepatitis A virus infection. *The American Journal of Medicine*.

[19] Ilan Y, Hillman M, Oren R, Zlotogorski A, Shouval D (1990). Vasculitis and cryoglobulinemia associated with persisting cholestatic hepatitis A virus infection. *The American Journal of Gastroenterology*.

[20] Yamaguchi M, Ohta A, Tsunematsu T (1992). Preliminary criteria for classification of adult Still's disease. *Journal of Rheumatology*.

[21] Seo S-R, Kim S-S, Lee S-J, Kim T-J, Park Y-W, Lee S-S (2011). Adult-onset still disease in a patient with acute hepatitis A. *Journal of Clinical Rheumatology*.

[22] Sridharan S, Mossad S, Hoffman G (2000). Hepatitis A infection mimicking adult onset Still's disease. *Journal of Rheumatology*.

[23] Willner IR, Uhl MD, Howard SC, Williams EQ, Riely CA, Waters B (1998). Serious hepatitis A: an analysis of patients hospitalized during an urban epidemic in the United States. *Annals of Internal Medicine*.

[24] Cuthbert JA (2001). Hepatitis A: old and new. *Clinical Microbiology Reviews*.

[25] Centers for Disease Control and Prevention (CDC) (2002). Positive test results for acute hepatitis a virus infection among persons with no recent history of acute hepatitis—United States. *Morbidity and Mortality Weekly Report*.

[26] Alatoom A, Ansari MQ, Cuthbert J (2013). Multiple factors contribute to positive results for hepatitis A virus immunoglobulin M antibody. *Archives of Pathology and Laboratory Medicine*.

